# Atrioventricular groove patch plasty in an infant with pulmonary stenosis after arterial switch operation: a case report

**DOI:** 10.1093/jscr/rjad052

**Published:** 2023-02-14

**Authors:** Hironobu Nishiori, Ikuo Hagino, Hiroshi Koshiyama, Takahiro Ito, Masaru Kumae, Mitsuru Aoki

**Affiliations:** Department of Cardiovascular Surgery, Chiba Children’s Hospital, Chiba City, Chiba, Japan; Department of Cardiovascular Surgery, Chiba Children’s Hospital, Chiba City, Chiba, Japan; Department of Cardiovascular Surgery, Chiba Children’s Hospital, Chiba City, Chiba, Japan; Department of Cardiovascular Surgery, Chiba Children’s Hospital, Chiba City, Chiba, Japan; Department of Cardiovascular Surgery, Chiba Children’s Hospital, Chiba City, Chiba, Japan; Department of Cardiovascular Surgery, Chiba Children’s Hospital, Chiba City, Chiba, Japan

**Keywords:** AV groove patch plasty, pulmonary stenosis, arterial switch, transposition of great artery

## Abstract

A 9-month-old infant developed pulmonary stenosis (PS) after an arterial switch operation for transposition of the great arteries, accompanied by a Shaher Type 4 coronary anatomy. As the right coronary artery (RCA) ran across the anterior side of the right ventricle (RV), atrioventricular (AV) groove patch plasty was performed to relieve PS. The distance between the RCA and tricuspid valve was confirmed by preoperative-computed tomography. The AV groove was carefully incised, ensuring the position of the tricuspid valve, and maintaining a distance of 3 mm from the tricuspid annulus to avoid approaching the RCA. While suturing the monocuspid valve patch, only the endocardial side of the RV was sutured, and RCA injury was prevented. Thus, especially in patients < 1 year of age, careful incision of the AV groove and suturing only the endocardial side is important to avoid injuring the RCA in AV groove patch plasty.

## INTRODUCTION

Pulmonary stenosis (PS) is a major complication after arterial switch operation (ASO) for the transposition of the great arteries (TGA). To relieve PS after ASO for TGA, right ventricular outflow tract repair (RVOTR) through an anterior right ventricular incision is not acceptable in cases with Shaher Type 4 coronary anatomy because it leads to right coronary artery (RCA) injury. In such cases, atrioventricular (AV) groove patch plasty has been reported as an optimal surgical option [1]. We successfully performed AV groove patch plasty in a 9-month-old infant to relieve PS after ASO. Herein, we report the methods and precautions used to avoid RCA injury in this surgical strategy for infants.

## CASE REPORT

**Figure 1:**
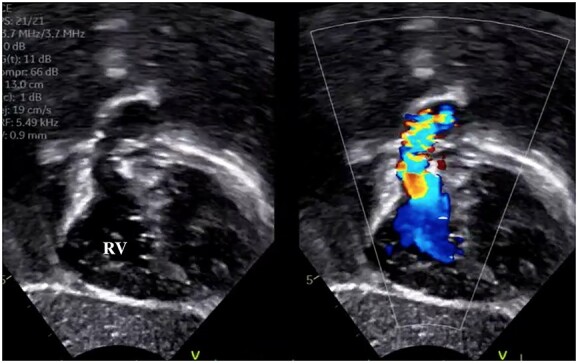
Preoperative transthoracic echocardiography showing right ventricular outflow tract narrowing and jet flow.

**Figure 2:**
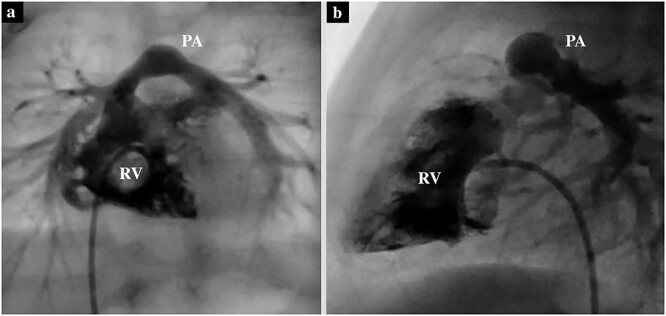
Preoperative cardiac catheterization of the axial view (**a**) and sagittal view (**b**) showing PS, with the pulmonary valve annulus diameter of 6.7 mm.

**Figure 3:**
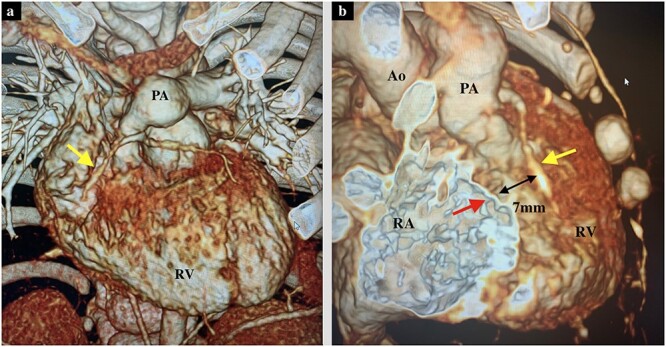
(**a**) Preoperative 3D-computed tomography of the axial view showing PS and the RCA (yellow arrow). (**b**) Preoperative 3D-computed tomography of the right oblique view showing the distance between tricuspid valve annulus (red arrow) and RCA (yellow arrow) of 7 mm. Ao, Aorta; RA, right atrium.

A male infant with TGA accompanied by Shaher Type 4 coronary anatomy underwent ASO at 5 days of age. Intraoperatively, the right ventricular outflow tract was injured during the RCA separation to obtain the mobilization and repaired using an autologous pericardial pledget, resulting in postoperative subvalvular PS. Follow-up evaluation showed relief of the subvalvular PS, but pulmonary valve stenosis, main pulmonary artery (PA) stenosis and right PA stenosis progressed because of the subvalvular jet. Transthoracic echocardiography showed a *trans*-right ventricle (RV) outflow peak velocity of 4.5 m/s, a systolic pressure gradient of 83 mmHg and a right ventricular fraction area change of 46.4% (Fig. 1). Cardiac catheterization showed a right ventricular pressure of 97 mmHg, a right ventricular/left ventricular pressure ratio of 0.99 and a systolic pressure gradient across the right ventricular outflow of 91 mmHg. The diameter of the pulmonary valve annulus was 6.1 mm (63.6%) (Fig. 2). Three-dimensional-computed tomography showed that the distance between the RCA and the tricuspid valve was 7 mm (Fig. 3).

**Figure 4:**
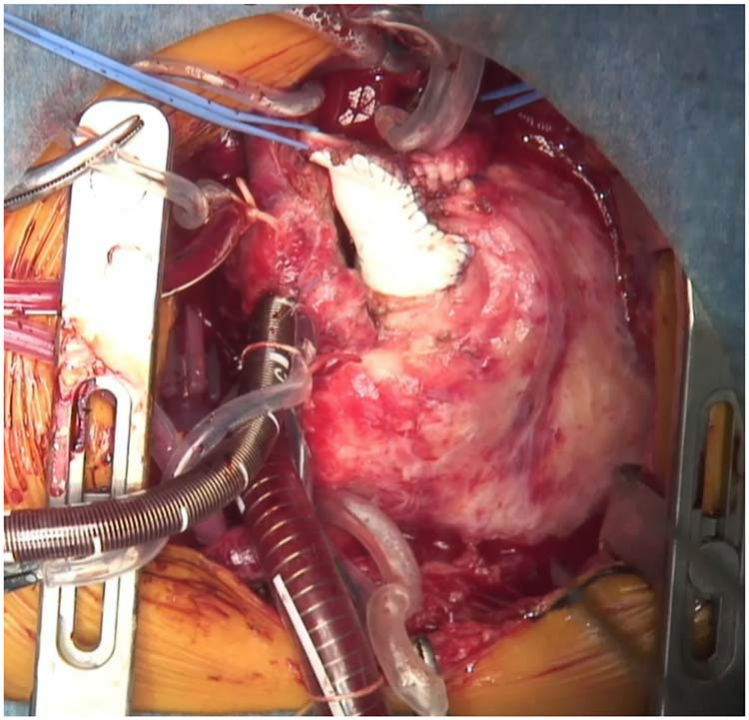
Intraoperative image of a monocuspid valve patch sutured to the PA and right ventricular outflow along the AV groove.

During the operation, the RCA could not be identified from the external surface. The main PA was incised longitudinally and extended into the right ventricular outflow tract along the AV groove between the RCA and the tricuspid annulus. The anterior leaflet of the tricuspid valve was identified during the right ventricular outflow incision, maintaining a distance of 3 mm from the tricuspid annulus to avoid approaching the RCA. The pulmonary cusps were highly fused, and pulmonary valve commissurotomy was performed. The right ventricular outflow tract was reconstructed using an expanded polytetrafluoroethylene (PTFE) monocuspid valve patch. While suturing the patch, only the endocardial side was sutured instead of all layers to avoid RCA injury (Fig. 4). The patient was discharged on postoperative Day 9. Six months post procedure, transthoracic echocardiography showed a *trans*-right ventricular outflow peak velocity of 1.4 m/s and moderate pulmonary valve regurgitation.

## DISCUSSION

PS is the most frequent complication after ASO, which occurs at a rate of 17–55%, and is reported to require surgical intervention in 1–22% of those affected [2, 3]. Factors associated with reoperation for PS after ASO are technical or anatomic; the major cause of PS is distortion or retraction of the pericardial patch used to cover the defect left by the coronary artery harvest [4]. When reoperation is required to relieve PS after ASO, RVOTR with a RV outflow incision can be performed in cases accompanied by several Shaher type anatomies. However, this procedure is not applicable to cases of PS after ASO for TGA accompanied by Shaher Type 3a, 3c, 3d, 4, 5c, 7c and 9 anatomies, since the RCA runs across the anterior side of the RV, and the incision can lead to RCA injury. AV groove patch plasty, a transannular patch repair using an ePTFE monocuspid valve patch incising along the AV groove, has been reported to be an ideal surgical option to relieve PS or pulmonary regurgitation after ASO for TGA Shaher type anatomy. This procedure was originally reported to relieve PS after ASO for anatomically modified macrovascular anomalies and was then applied to {S, D, L} type double outlet RV and corrected TGA [1, 5].

PS can occur within the first year after ASO, and the risk of reoperation for PS remains high for nine years post procedure [4]. Morita *et al*. [5] and Hiramatsu *et al*. [1] have reported favorable outcomes of AV groove patch plasty in patients ranging in age from 2 to 34 years old. As, in our case, the patient was 9 months of age, it was important to identify the detailed anatomy around the AV groove. Preoperative CT was a useful modality to confirm the location and distance between the tricuspid valve and RCA. It has been reported that AV groove patch plasty is safe when the distance between the incision along the right AV groove and the RCA is more than 5 mm [1]. However, in this case, the distance between the right ventricular incision and the RCA was only 3 mm. It was important to make a right ventricular incision along the tricuspid valve, suturing and stitching only the endocardial side, not all layers, to avoid injuring the RCA.

The Rastelli procedure using a conduit is another option to relieve PS after ASO. However, if the Rastelli procedure is performed after ASO, the 12–14 mm conduit will be retrosternal, which leads to compression of the conduit by the sternum. This consideration made the Rastelli procedure unsuitable for this case. AV groove patch plasty is a great alternative surgical strategy; however, there is concern regarding progression of pulmonary regurgitation. Periodic follow-up echocardiography is necessary to assess both pulmonary regurgitation progression and right ventricular function. In cases where revision becomes necessary, redo AV groove patch plasty should be performed, or, once sufficient growth has been achieved, pulmonary valve replacement becomes a therapeutic option.

AV groove patch plasty is a great therapeutic option for PS developing after ASO for TGA accompanied by a Shaher Type 4 or 9 anatomy. Careful incision of the AV groove and suturing only the endocardial side is important to avoid injuring the RCA, especially in patients less than one year of age.
